# Investigation of the nutritional and functional roles of a combinational use of xylanase and β-glucanase on intestinal health and growth of nursery pigs

**DOI:** 10.1186/s40104-024-01021-8

**Published:** 2024-05-05

**Authors:** Hyunjun Choi, Yesid Garavito Duarte, Guilherme A. M. Pasquali, Sung Woo Kim

**Affiliations:** 1 Department of Animal Science, North Carolina State University, 116 Polk Hall, Campus Box 7621, Raleigh, NC 27695 USA; 2 BASF SE, Ludwigshafen, 67056 Germany

**Keywords:** Glucanase, Growth performance, Intestinal health, Nursery pigs, Xylanase

## Abstract

**Background:**

Xylanase and β-glucanase combination (XG) hydrolyzes soluble non-starch polysaccharides that are anti-nutritional compounds. This study aimed to evaluate the effects of increasing levels of XG on intestinal health and growth performance of nursery pigs.

**Methods:**

Forty pigs (6.5 ± 0.4 kg) were assigned to 5 dietary treatments and fed for 35 d in 3 phases (11, 9, and 15 d, respectively). Basal diets mainly included corn, soybean meal, and corn distiller’s dried grains with solubles, contained phytase (750 FTU/kg), and were supplemented with 5 levels of XG at (1) 0, (2) 280 TXU/kg xylanase and 125 TGU/kg β-glucanase, (3) 560 and 250, (4) 840 and 375, or (5) 1,120 and 500, respectively. Growth performance was measured. On d 35, all pigs were euthanized and jejunal mucosa, jejunal digesta, jejunal tissues, and ileal digesta were collected to determine the effects of increasing XG levels and XG intake on intestinal health.

**Results:**

Increasing XG intake tended to quadratically decrease (*P* = 0.059) viscosity of jejunal digesta (min: 1.74 mPa·s at 751/335 (TXU/TGU)/kg). Increasing levels of XG quadratically decreased (*P* < 0.05) Prevotellaceae (min: 0.6% at 630/281 (TXU/TGU)/kg) in the jejunal mucosa. Increasing XG intake quadratically increased (*P* < 0.05) Lactobacillaceae (max: 40.3% at 608/271 (TXU/TGU)/kg) in the jejunal mucosa. Increasing XG intake quadratically decreased (*P* < 0.05) Helicobacteraceae (min: 1.6% at 560/250 (TXU/TGU)/kg) in the jejunal mucosa. Increasing levels of XG tended to linearly decrease (*P* = 0.073) jejunal IgG and tended to quadratically increase (*P* = 0.085) jejunal villus height to crypt depth ratio (max: 2.62 at 560/250 (TXU/TGU)/kg). Increasing XG intake tended to linearly increase the apparent ileal digestibility of dry matter (*P* = 0.087) and ether extract (*P* = 0.065). Increasing XG intake linearly increased (*P* < 0.05) average daily gain.

**Conclusions:**

A combinational use of xylanase and β-glucanase would hydrolyze the non-starch polysaccharides fractions, positively modulating the jejunal mucosa-associated microbiota. Increased intake of these enzyme combination possibly reduced digesta viscosity and humoral immune response in the jejunum resulting in improved intestinal structure, and ileal digestibility of nutrients, and finally improving growth of nursery pigs. The beneficial effects were maximized at a combination of 550 to 800 TXU/kg xylanase and 250 to 360 TGU/kg β-glucanase.

## Background

The use of alternative feedstuffs such as corn distiller’s dried grains with solubles (DDGS) has been dramatically increased due to the availability and economic advantages [1]. A notable issue arises with the increased use of alternative feedstuffs primarily due to high contents of non-starch polysaccharides (NSP), including arabinoxylans and β-glucans, negatively affecting the intestinal health and growth of nursery pigs [2–4]. The NSP, once ingested, leads to an increase in bulkiness [5, 6] and viscosity of the digesta, mainly caused by the soluble fraction of NSP inhibiting endogenous enzymes from accessing feed particles in the small intestine, subsequently reducing nutrient utilization in pigs [2, 7]. Increased digesta viscosity in the small intestine can increase the risk of pathogenic bacterial overgrowth as it slows down the digesta passage rate and accelerates the proliferation of ammonia-producing bacteria from the fermentation of digesta [8, 9]. According to previous studies, mucosa-associated microbiota has direct correlation with intestinal health of the host animals compared with luminal microbiota in pigs [10, 11] and the composition of luminal microbiota is distinctly different from that of mucosa-associated microbiota [12], suggesting the importance of investigating mucosa-associated microbiota in relation to the host intestinal health.

Xylanase supplementation in feeds increases the hydrolysis of arabinoxylans, reducing digesta viscosity [3, 13] and releasing xylooligosaccharides in the small intestine of pigs [14, 15], which exhibit potential prebiotic effects [16, 17]. In addition, β-glucanase hydrolyzes cereal β-glucans by cleaving β-glycosidic linkages into glucooligosaccharides [18, 19], which can positively modulate intestinal microbiota [20, 21]. Individual NSP degrading enzyme supplementation, including xylanase or β-glucanase, has been shown to modulate the diversity and relative abundance of intestinal microbiota in animals [13, 20, 22], potentially by releasing oligosaccharides fractions and reducing digesta viscosity in the small intestine of pigs [3, 23, 24]. Phytase has been extensively used in pig feeds to release entrapped nutrients, including minerals and energy bound in the form of phytate [25, 26]. Phytase also releases proteins, non-selectively bound with phytate, improving enzyme activity, such as trypsin in the small intestine in animals [27, 28], potentially altering the nutrient utilization and intestinal microbiota of pigs [29].

Considering the complex nature of NSP fractions and structures, the combinational use of feed enzymes including NSP-degrading enzymes and phytase has shown synergistic effects on the hydrolysis of NSP and phytate and the release of nutrients entrapped by NSP [30, 31], positively affecting the intestinal microbiota [20], nutrient utilization [31], and growth of pigs [3, 32, 33]. Based on previous findings, this study aimed to test the hypothesis that a combinational use of xylanase and β-glucanase in pig feeds with phytase would have positive impacts on the composition of intestinal microbiota and intestinal health with reduced digesta viscosity, reduced excessive immune reactions, improved feed digestibility, and finally improved growth of nursery pigs. To test these hypotheses, the objective of this study was to evaluate the intestinal health and growth performance of nursery pigs affected by dietary supplementation of increasing levels of combinational use of xylanase and β-glucanase.

## Materials and methods

The protocol of this experiment was reviewed and approved by North Carolina State University Animal Care and Use Committee (Raleigh, NC, USA).

### Animals, experimental design, and experimental diets

Forty newly weaned pigs (20 barrows and 20 gilts) at 21 days of age with initial body weight (BW) at 6.5 ± 0.4 kg were assigned to 5 dietary treatments in a randomized complete block design with initial BW and sex as block factors. Each treatment had 8 replicates (4 pens with barrows and 4 pens with gilts). Pigs were individually housed in pens (1.50 m × 0.74 m) and had free access to feeds and water throughout the experimental period. Basal diets mainly included corn, soybean meal, and corn DDGS with phytase (750 FTU/kg) supplemented with 5 levels of xylanase and β-glucanase combination (XG; Natugrain TS, BASF SE, Germany) at (1) 0, (2) 280 TXU/kg xylanase and 125 TGU/kg β-glucanase, (3) 560 TXU/kg and 250 TGU/kg, (4) 840 TXU/kg and 375 TGU/kg, or (5) 1,120 TXU/kg and 500 TGU/kg, respectively (Table 1). Experimental diets were formulated to meet or exceed the nutrient requirements suggested by NRC (2012) [34], except for Ca and P in 3 phases: phase 1 (d 0 to 11), phase 2 (d 11 to 20), and phase 3 (d 20 to 35). Total Ca and standardized total tract digestible P in experimental diets were 0.17% and 0.10%, respectively, lower in comparison to the recommendations from NRC (2012) [34] to account for phytase effects [35]. The 750 FTU/kg of phytase in feeds was supplemented following a standard inclusion levels with high vitamin and mineral contents in nursery pig feeds [36]. The experimental diets were provided as mash form. The experimental diets did not contain Zn and Cu at pharmacological levels, and antibiotics were not included in feeds as a growth promoter. Activities of xylanase and β-glucanase in feeds were measured and analyzed enzyme activities in the feeds are described in Table 1. One unit of endo-1,4-β-xylanase activity (TXU) is defined as the amount of enzyme required to liberate 5 μmol of xylose per min at 40 °C in 100 mL buffer solution containing 1 g of arabinoxylan, pH 3.5. One unit of endo-1,4-β-glucanase activity (TGU) is defined as the amount of enzyme required to liberate 1 μmol of glucose per min at 40 °C in 100 mL buffer solution containing 1 g of β-glucan, pH 3.5. Titanium dioxide (TiO_2_) was included at 0.40% to the phase 3 diets as an indigestible marker to determine the apparent ileal digestibility (AID) of nutrients and energy.

**Table 1:** Composition of basal diets (as-fed basis)

Item	Phase 1	Phase 2	Phase 3
Feedstuff, %			
Corn (yellow dent)	41.44	46.48	51.12
Soybean meal (48% CP)	17.00	15.50	14.00
Corn distiller’s dried grains with solubles	5.00	15.00	30.00
Whey permeate	20.00	12.00	0.00
Processed soybean meal^a^	6.00	3.00	0.00
Blood plasma	6.00	3.00	0.00
Poultry fat	0.97	1.22	0.62
L-Lys HCl	0.44	0.60	0.77
L-Met	0.19	0.17	0.13
L-Thr	0.13	0.16	0.19
L-Trp	0.00	0.02	0.05
L-Val	0.03	0.05	0.04
L-Ile	0.01	0.02	0.01
Dicalcium phosphate	0.10	0.10	0.10
Limestone	1.30	1.29	1.19
Vitamin premix^b^	0.03	0.03	0.03
Mineral premix^c^	0.15	0.15	0.15
Sodium chloride	0.22	0.22	0.22
Enzyme mixture^d^ (corn and feed enzyme)	0.50	0.50	0.50
Phytase mixture^e^ (corn and phytase)	0.50	0.50	0.50
Titanium dioxide	0.00	0.00	0.40
Calculated composition			
Dry matter, %	91.53	90.62	89.29
Metabolizable energy, kcal/kg	3,400	3,400	3,350
Crude protein, %	22.07	20.53	20.21
SID^f^ Lys, %	1.50	1.35	1.23
SID Met + Cys, %	0.82	0.74	0.68
SID Thr, %	0.88	0.79	0.73
SID Trp, %	0.26	0.22	0.20
SID Val, %	0.95	0.86	0.78
Total Ca, %	0.68	0.63	0.53
STTD^g^ P, %	0.36	0.30	0.23
Total P, %	0.54	0.48	0.43
Analyzed composition			
Dry matter, %	89.54	88.74	87.83
Gross energy, kcal/kg	-	-	4,012
Crude protein, %	21.45	20.12	19.35
Neutral detergent fiber, %	6.23	9.91	12.43
Acid detergent fiber, %	3.13	3.63	5.80
Ether extract, %	3.26	4.04	4.07

^a^Processed soybean meal was a hydrolyzed soy protein product (HP 300) from Hamlet Protein (Findlay, OH, USA)

^b^The trace mineral premix provided per kilogram of complete diet: 33 mg of Mn as manganese oxide, 110 mg of Fe as ferrous sulfate, 110 mg of Zn as zinc sulfate, 16.5 mg of Cu as copper sulfate, 0.30 mg of I as ethylenediamine dihydroiodide, and 0.30 mg of Se as sodium selenite

^c^The vitamin premix provided per kilogram of complete diet: 6,614 IU of vitamin A as vitamin A acetate, 992 IU of vitamin D_3_, 19.8 IU of vitamin E, 2.64 mg of vitamin K as menadione sodium bisulfate, 0.03 mg of vitamin B_12_, 4.63 mg of riboflavin, 18.52 mg of D-pantothenic acid as calcium pantothenate, 24.96 mg of niacin, and 0.07 mg of biotin

^d^Five dietary treatments consisted of 5 levels of NSP degrading enzyme with xylanase and β-glucanase combination (XG: 0, 280/125, 560/250, 840/375, and 1,120/500 (TXU/TGU)/kg) supplemented to basal diets; XG were supplemented at the expense of corn in the enzyme mixture; the analyzed activities of xylanase and β-glucanase in feeds were 65/44, 360/193, 637/288, 775/424, and 1,114/466 (TXU/TGU)/kg in phase 1, 121/194, 421/267, 682/445, 936/458, and 1,226/517 (TXU/TGU)/kg in phase 2, and 89/153, 424/272, 561/428, 987/562, and 1,340/708 (TXU/TGU)/kg in phase 3, respectively

^e^Phytase supplementation level was 750 FTU/kg in basal diets

^f^*SID* Standardized ileal digestible

^g^*STTD* Standardized total tract digestible

### Sample and data collection

The BW of pigs and feed disappearance were measured on d 0, 11, 20, and 35 to determine average daily gain (ADG), average daily feed intake (ADFI), and gain to feed ratio (G:F) for growth performance of pigs [37]. Fecal scores of each pen were recorded based on a 1 to 5 scale (1: firm and 5: watery) by visual observation of fresh feces from d 3 at 2-day intervals [3, 38] as pigs did not defecate until d 3. At d 35, all pigs were euthanized by the penetration of a captive bolt followed by exsanguination. After euthanasia, jejunal mucosa, jejunal tissues, jejunal digesta, and ileal digesta were collected. Mid-jejunum tissues were obtained from 3 to 4 m after the pyloric valve of stomach of pigs. The mid-jejunal tissues (20 cm) were flushed with 0.9% saline solution to remove jejunal digesta. The first 15 cm was used to collect jejunal mucosa by scraping the mucosa layer in the jejunum using a glass microscope slide and the remaining 5 cm was fixed in 10% buffered formaldehyde to be used for Ki-67 staining and histological evaluation [39]. Jejunal mucosa were collected for tumor necrosis factor-alpha (TNF-α), interleukin-8 (IL-8), immunoglobulin A (IgA), and immunoglobulin G (IgG) as indicators of immune response status and protein carbonyl and malondialdehyde (MDA) as indicators of oxidative damage products. Jejunal digesta samples were collected into 15-mL tubes, kept on ice, and viscosity was measured on the sampling date, after collection. The jejunal mucosal samples were transferred to the freezer at −80 °C for further process and analysis including DNA extraction, immune responses, and oxidative damage products. Ileal digesta was collected in a 50-mL container and stored at −20 °C for further process and analysis to determine the AID of nutrients and energy.

### Viscosity of jejunal digesta

Viscosity of jejunal digesta was measured using a Brookfield digital viscometer (Model DV-II Version 2.0, Brookfield Engineering Laboratories Inc., Stoughton, MA, USA). The 15-mL tubes containing jejunal digesta were centrifuged at 1,000 × *g* at 4 °C for 10 min to obtain the liquid phase for supernatant. After the first centrifuging process, the liquid phase was transferred to a 2-mL tube to centrifuge at 10,000 × *g* at 4 °C for 10 min. The supernatant was transferred to another 2-mL tube for further measurement. The 0.5 mL of centrifuged jejunal digesta were placed in the viscometer set at 25 °C. Viscosity measurement was the average between 45.0/s and 22.5/s shear rates, and the viscosity were recorded as apparent viscosity in millipascal seconds (mPa·s). The viscosity was measured 3 times per jejunal digesta sample with 2 internal replications.

### Diversity and relative abundance of mucosa-associated microbiota in the jejunum

Four jejunal mucosa representing the approximate median BW of each treatment were selected for mucosa-associated microbiota analysis. The jejunal mucosa were sent to Zymo Research Corporation (Irvine, CA, USA) to evaluate diversity and relative abundance of mucosa-associated microbiota in the jejunum. Jejunal mucosa were used for DNA extraction using the ZymoBIOMICS-96 MagBead DNA kit (Zymo Research). The extracted DNA samples were prepared for 16S RNA sequencing with the Quick-16S Primer Set V3-V4 (Zymo Research) and NGS library Preparation kits for microbial analysis. These primers are custom-designed by Zymo Research to provide the best coverage of the 16S gene. The final PCR products were quantified with qPCR fluorescence readings and pooled together based on equal molarity. The final pooled library was cleaned up with the Select-a-Size DNA Clean & Concentrator (Zymo Research), then quantified with TapeStation (Agilent Technologies, Santa Clara, CA, USA) and Qubit (Thermo Fisher Scientific, Waltham, WA, USA). For sequencing, the final library was sequenced on Illumina NextSeq 2000 with a P1 (Cat. 20075294) reagent kit (600 cycles). The sequencing was performed with 30% PhiX spike-in. Taxonomy assignment was performed using Uclust from Qiime v.1.9.1. Taxonomy was assigned with the Zymo Research Database, a 16S database that is internally designed and curated, as reference. Alpha diversity rare fraction plot generation and the amplicon sequence variant (ASV) table generation were performed with Qiime (version 1.9.1) [40]. The depth of sequencing coverage was > 20,000 × sample. The ASV data were transformed to relative abundance for further statistical analysis, and the ASV data with less than 0.5% abundance within each level were combined as “others”.

### Immune responses and oxidative damage products in the jejunum

One gram of jejunal mucosa sample was weighed and ground using a homogenizer (Tissuemiser, Thermo Fisher Scientific Inc., Rockford, IL, USA) on ice in 2 mL phosphate-buffered saline for 30 s. The homogenate was centrifuged at 14,000 × *g*, at 4 °C for 30 min to obtain supernatant, which was used to determine the contents of total protein, IgA, IgG, TNF-α, IL-8, protein carbonyl, and MDA. The supernatant was pipetted off and kept at −80 °C. The content of total protein of mucosa was determined using the kit Pierce BCA Protein Assay (23225#, Thermo Fisher Scientific Inc.) to calculate the contents of IgA, IgG, TNF-α, IL-8, protein carbonyl, and MDA per milligram of protein in the jejunal mucosa sample. The contents of IgA and IgG were analyzed using ELISA kits for pig IgA (E101-102, Bethyl Laboratories, Inc., Montgomery, TX, USA) and pig IgG (E101-104, Bethyl Laboratories, Inc.), respectively. The mucosal samples were diluted to 1:1,000 and 1:1,600 with PBS to analyze IgA and IgG, respectively. The contents of MDA and protein carbonyl were measured by commercial kits (Cell Biolabs, Inc., San Diego, CA, USA) following the protocols of the manufacturer. The contents of TNF-α and IL-8 in jejunal mucosa were measured by ELISA kits (R&D Systems, Minneapolis, MN, USA) following Deng et al. [41].

### Intestinal morphology and crypt cell proliferation in the jejunum

After 48 h in 10% buffered formaldehyde solution, two sections of the jejunum per pig were transversely cut, placed into a cassette in 70% ethanol, and sent to the University of North Carolina Histology Laboratory (UNC School of Medicine, Chapel Hill, NC, USA) for dehydration, embedment, and Ki-67^+^ immunohistochemistry staining for morphological evaluation and to evaluate cell proliferation in the crypt. Pictures of villus and crypts in 40 × magnification were taken and measured villus height (VH) and crypt depth (CD) using a camera Infinity 2-2 digital CCD attached to a microscope Olympus CX31 (Lumenera Corporation, Ottawa, Canada) for intestinal morphology. The VH to CD ratio (VH:CD) was also determined. Pictures of crypts in 100 × magnification were taken for Ki-67^+^ cell measurement. The ImageJS software was used for calculating the percentage of dyed Ki-67^+^ cells in the total cells in the crypt. The percentage of Ki-67^+^ cells was used as an indicator of enterocyte proliferation in the crypt. All analyses of the morphology were executed by the same person, and the average 15 measurements of each sample were calculated and reported as one number per sample.

### Chemical analysis

Frozen ileal digesta were dried in a freeze-drier. Experimental diets and dried ileal digesta were finely ground and dried in the forced-air drying oven at 135 °C for 2 h to determine dry matter (DM; method 930.15), and ether extract (EE) was analyzed using anhydrous diethyl ether (method 920.39) as described in AOAC [42]. Nitrogen content in diets and the ileal digesta was measured using a TrueSpec N Nitrogen Determinator (LECO Corp., St. Joseph, MI, USA) to determine crude protein (CP, 6.25 × N). Experimental diets and the ileal digesta were analyzed for gross energy (GE) using bomb calorimetry (Parr 1261, Parr Instrument Co., Moline, IL, USA), detecting energy released during the complete combusion of a sample. The diets were analyzed for neutral detergent fiber (method 2002.04) and acid detergent fiber (method 973.18) as described in AOAC [42], using an ANKOM 200 Fiber Analyzer (Ankom Technology Corp., Macedon, NY, USA). The xylanase activity in feeds was measured by quantifing reducing sugar equipvalent (xylose or glucose) after incubation in 0.05 mol/L sodium acetate buffer (pH 5.3) at 50 °C for 60 min and used colorimetic assay at 540 nm following Inborr et al. [43] and Moita et al. [13]. Dietary β-glucanase activity was measured using a β-glucanase commercial assay kit (Megazyme, Ireland) with modifications in the amounts of samples following Duarte et al. [20] and de Brito et al. [44].

### Apparent ileal digestibility of nutrients and energy

The TiO_2_ concentrations of the diets and ileal digesta were determined [45, 46]. Briefly, the samples were digested in Kjeldahl digestion tubes with a catalyst and 13 mL of concentrated sulfuric acid at 420 °C for 2 h. After cooling for 30 min, 10 mL of 30% hydrogen peroxide was added each tube, and total liquid volume was adjusted to 100 mL with distilled water. The liquid was transferred to a microplate to determine TiO_2_ at 410 nm using spectrophotometry. The AID of GE was calculated according to the following equation:

$$\mathrm{AID\,of\,GE},\mathrm{\,\%}=[1-({{\text{TiO}}}_{2{\text{diet}}}\,/\,{{\text{TiO}}}_{2{\text{digesta}}})\times ({{\text{GE}}}_{{\text{digesta}}}\,/\,{{\text{GE}}}_{{\text{diet}}})]\times\,100$$

where TiO_2diet_ and TiO_2digesta_ are the TiO_2_ contents in the diet and ileal digesta, respectively (%; DM basis); and GE_digesta_ and GE_diet_ are the energy contents in the ileal digesta and diet, respectively (kcal/kg; DM basis). The AID of nutrients (DM, CP, and EE) were also calculated using the same equation. Nutrient contents (DM basis) were expressed as %.

### Statistical analyses

Experimental data were analyzed using the MIXED procedure (SAS Inst., Cary, NC, USA). The statistical model included dietary treatment as a fixed effect and initial BW and sex as random effects. A power test was conducted to determine the number of replications needed for this study. To determine the statistical significance of expected mean difference of 7% to 8% at *P* < 0.05, considering 5% coefficient of variation based on previous studies with pigs of similar genetic background and under similar research environment [13, 47], and with a desired power of test (1 – beta) set at 95%, the power analysis indicated an 80% power. Based on this analysis, the minimum number of replications each treatment was determined to be 8 [48]. One observation of a pig fed a diet with 560/250 (TXU/TGU)/kg was removed from the data set for the final analysis as the observation deviated by more than 1.5 times the interquartile ranges from the treatment median value of growth performance, showing inability to eat. The least squares mean of each treatment was calculated. The effects of increasing levels of XG in the diets of nursery pigs were determined using the polynomial contrasts (linear and quadratic effects) with coefficients by the Proc IML procedure of SAS 9.4. The XG intake ((TXU/TGU)/d) was calculated to ADFI (g/d) during the overall period multiplied by the level of XG ((TXU/TGU)/kg) to determine the effects of increasing XG intake on jejunal digesta viscosity, jejunal mucosa-associated microbiota, immune responses, intestinal morphology, nutrient digestibility, and growth performance of nursery pigs. The linear and quadratic effects of XG intake on the parameters were investigated by the RSREG procedure. For the optimal levels of XG (%), the XG level for reaching the maximum or minimum value of the parameter was converted from XG intake (TXU/d feed) by dividing with the overall average daily feed intake (0.664 kg/d). The experimental unit was a pen. The statistical significance and tendency were declared at *P* < 0.05 and 0.05 ≤ *P* < 0.10, respectively.

## Results

### Enzyme activities in experimental diets

Increasing levels of XG ranged from 65/44 to 1,114/466 (TXU/TGU)/kg in phase 1, 121/194 to 1,226/517 (TXU/TGU)/kg in phase 2, and 89/153 to 1,340/708 (TXU/TGU)/kg in phase 3, respectively (Table 1) and the analyzed activities were similar to calculated activities.

### Viscosity of jejunal digesta

**Fig. 1:**
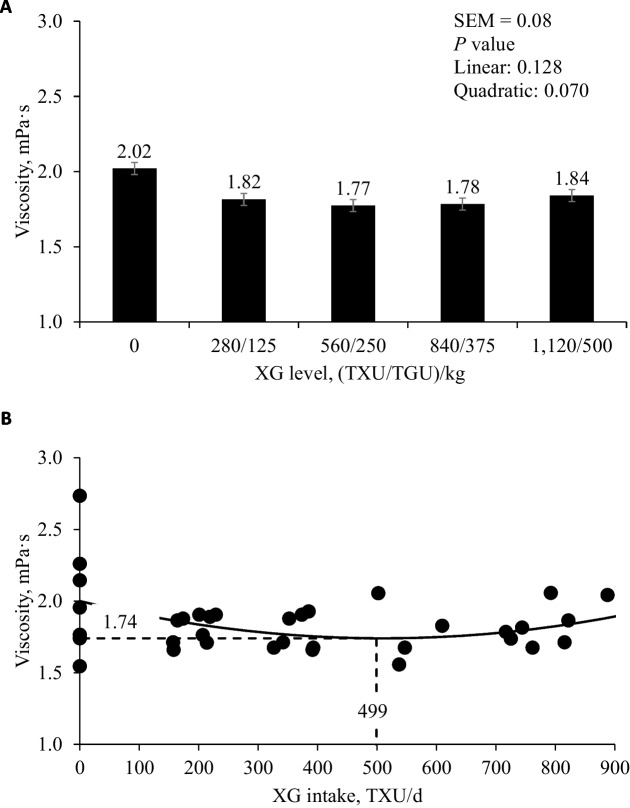
**A** Changes in the viscosity of jejunal digesta (as-is basis) in nursery pigs fed diets with increasing levels of xylanase and β-glucanase combination (XG). Each level had 8 replicates. **B** Viscosity of jejunal digesta in nursery pigs fed diets with increasing levels of xylanase and β-glucanase combination (XG) intake (TXU/d). Viscosity, mPa·s = 0.000001 × XG intake × XG intake − 0.00102 × XG intake + 2.00 (Min: 1.74 at 499 TXU/d); *P* value of overall model: 0.059, *P* value of intercept: < 0.001, and *P* value of XG intake × XG intake: 0.041*; P* value of XG intake: 0.022. The XG level for reaching the minimum value of viscosity was 751/335 (TXU/TGU)/kg converted from XG intake (499/223 TXU/d feed) by dividing with the overall average daily feed intake (0.664 kg/d). The number of observation was 40

Increasing levels of XG tended to quadratically decrease (*P* = 0.070) viscosity of jejunal digesta (Fig. 1). Increasing XG intake tended to quadratically decrease (*P* = 0.059) viscosity of jejunal digesta reaching the minimum (1.74 mPa·s) at 751 TXU/kg and 335 TGU/kg.

### Diversity and relative abundance of mucosa-associated microbiota in the jejunum

**Fig. 2:**
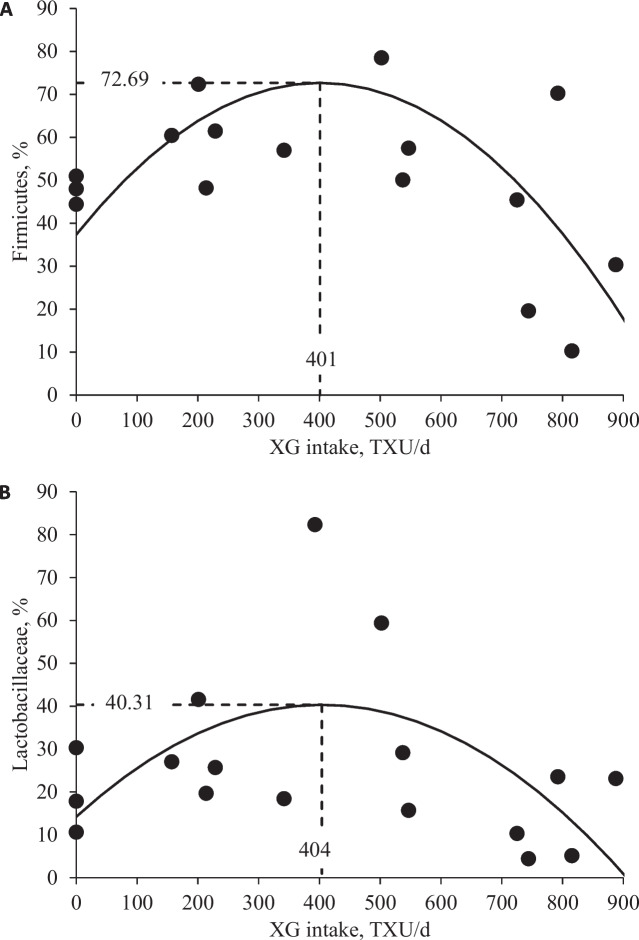
**A** Relative abundance of Firmicutes in the jejunal mucosa of nursery pigs fed diets with increasing levels of xylanase and β-glucanase combination (XG) intake (TXU/d). Firmicutes abundance, % = −0.00022 × XG intake × XG intake − 0.1763 × XG intake + 37.37 (Max: 72.69 at 401 TXU/d); *P* value of overall model: < 0.05, *P* value of intercept: < 0.05, and *P* value of XG intake × XG intake: < 0.05*; P* value of XG intake: < 0.05. The XG level for reaching the maximum value of Firmicutes was 604/270 (TXU/TGU)/kg converted from XG intake (401/179 (TXU/TGU)/d feed) by dividing with the overall average daily feed intake (0.664 kg/d). **B** Relative abundance of Lactobacillaceae in the jejunal mucosa of nursery pigs fed diets with increasing levels of xylanase and β-glucanase combination (XG) intake (TXU/d). Lactobacillaceae abundance, % = −0.00016 × XG intake × XG intake + 0.12925 × XG intake + 14.21 (Max: 40.31 at 404 TXU/d); *P* value of overall model: 0.095, *P* value of intercept: 0.196, and *P* value of XG intake × XG intake: < 0.05*; P* value of XG intake: < 0.05. The XG level for reaching the maximum value of Lactobacillaceae was 608/271 (TXU/TGU)/kg converted from XG intake (404/180 (TXU/TGU)/d feed) by dividing with the overall average daily feed intake. The number of observation was 20

**Fig. 3:**
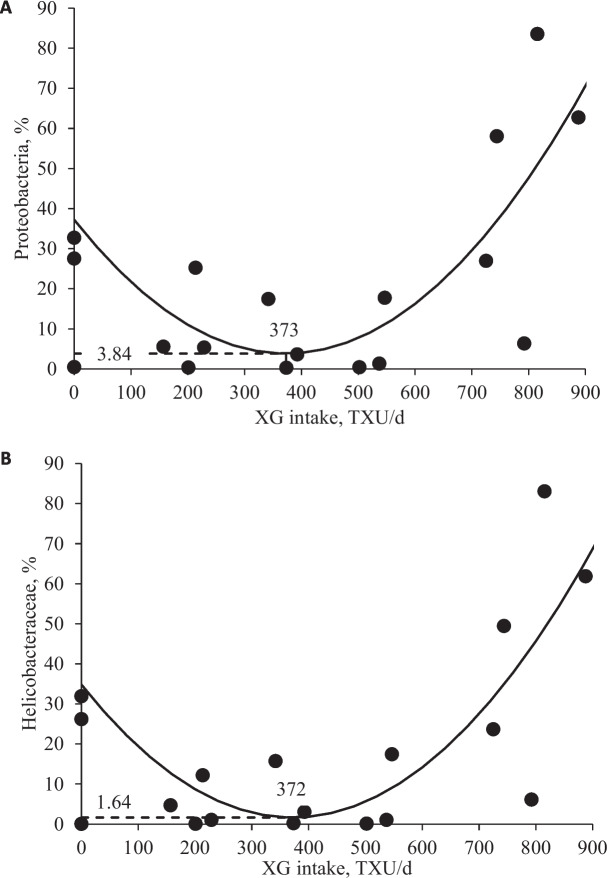
**A** Relative abundance of Proteobacteria in the jejunal mucosa of nursery pigs fed diets with increasing levels of xylanase and β-glucanase combination (XG) intake (TXU/d). Proteobacteria abundance, % = 0.00024 × XG intake × XG intake − 0.1825 × XG intake + 34.76 (Min: 3.84 at 373 TXU/d); *P* value of overall model: < 0.05, *P* value of intercept: < 0.05, and *P* value of XG intake × XG intake: < 0.05*; P* value of XG intake: < 0.05. The XG level for reaching the minimum value of Proteobacteria was 562/251 (TXU/TGU)/kg converted from XG intake (373/167 (TXU/TGU)/d feed) by dividing with the overall average daily feed intake (0.664 kg/d). **B** Relative abundance of Helicobacteraceae in the jejunal mucosa of nursery pigs fed diets with increasing levels of xylanase and β-glucanase combination (XG) intake (TXU/d). Helicobacteraceae abundance, % = 0.00024 × XG intake × XG intake − 0.17845 × XG intake + 34.81 (Min: 1.64 at 372 TXU/d); *P* value of overall model: < 0.05, *P* value of intercept: < 0.05, and *P* value of XG intake × XG intake: < 0.05*; P* value of XG intake: < 0.05. The XG level for reaching the minimum value of Helicobacteraceae was 560/250 (TXU/TGU)/kg converted from XG intake (372/166 (TXU/TGU)/d feed) by dividing with the overall average daily feed intake. The number of observation was 20

Increasing levels of XG tended to quadratically increase (*P* = 0.098) Simpson index of mucosa-associated microbiota in the jejunal mucosa, indicating an increase in species diversity within a specific ecological community (Table 2). However, there were no differences in Chao 1 and Shannon indexes of jejunal mucosa-associated microbiota with increasing levels of XG, indicating no increase in species richness or species diversity within the community, respectively. Increasing levels of XG quadratically decreased (*P* < 0.05) Bacteroidetes and Proteobacteria and quadratically decreased (*P* < 0.05) Prevotellaceae in the jejunal mucosa (Table 3). Increasing levels of XG quadratically increased Firmicutes (*P* < 0.05) and Lactobacillaceae (*P* < 0.05), respectively. Increasing levels of XG quadratically decreased (*P* < 0.05) Helicobacteraceae. Increasing levels of XG quadratically increased *Lactobacillus* (*P* < 0.05) and quadratically decreased *Helicobacter* (*P* < 0.05) and *Helicobacter rappini* (*P* < 0.05; Tables 4 and 5). Increasing XG intake quadratically increased (*P* < 0.05) Firmicutes and Lactobacillaceae in the jejunal mucosa (Fig. 2). Increasing XG intake quadratically decreased (*P* < 0.05) Proteobacteria and Helicobacteraceae in the jejunal mucosa (Fig. 3).

**Table 2:** Alpha diversity of jejunal mucosa-associated microbiota at the species level in nursery pigs fed diets with increasing levels of xylanase and β-glucanase combination (XG)^a^

Item	XG, (TXU/TGU)/kg					SEM^b^	*P* value	
	0	280/125	560/250	840/375	1,120/500		Linear	Quadratic
Chao 1	268.9	290.0	402.0	233.6	232.6	91.7	0.663	0.360
Shannon	4.37	5.37	5.28	4.56	4.20	0.79	0.642	0.263
Simpson	0.74	0.94	0.91	0.87	0.75	0.10	0.906	0.098

^a^Experimental unit was a pig and each level had 4 replicates (totaling 20 observations), representing the approximate median BW of each level

^b^*SEM* Standard error of the means

**Table 3:** Relative abundance of jejunal mucosa-associated microbiota at the phylum and family level in nursery pigs fed diets with increasing levels of xylanase and β-glucanase combination (XG)^a^

Item	XG, (TXU/TGU)/kg					SEM^b^	*P* value	
	0	280/125	560/250	840/375	1,120/500		Linear	Quadratic
Phylum								
Firmicutes	36.95	60.61	80.70	51.42	39.08	10.27	0.881	0.007
Proteobacteria	37.84	9.13	7.14	19.39	44.89	13.32	0.572	0.027
Actinobacteria	16.12	23.88	9.10	22.99	10.50	7.72	0.627	0.689
Bacteroidetes	7.72	0.91	2.14	1.40	4.14	2.11	0.336	0.048
Others	1.37	5.47	0.93	4.80	1.39	2.96	0.947	0.560
Family								
Lactobacillaceae	14.91	29.01	50.09	27.69	14.99	11.87	0.975	0.043
Helicobacteraceae	36.09	4.49	6.40	17.01	43.67	12.54	0.497	0.018
Bifidobacteriaceae	13.42	19.40	5.99	19.59	7.93	6.67	0.616	0.745
Streptococcaceae	2.88	9.03	6.78	3.70	3.29	2.86	0.536	0.124
Erysipelotrichaceae	2.79	7.91	3.70	5.28	6.15	2.62	0.625	0.784
Lachnospiraceae	4.71	3.56	6.22	4.40	2.73	2.21	0.628	0.478
Veillonellaceae	4.13	3.95	4.18	4.49	4.22	1.46	0.880	0.986
Prevotellaceae	7.14	0.85	1.80	1.34	3.95	1.96	0.358	0.042
Ruminococcaceae	3.77	0.93	4.56	1.89	1.49	1.55	0.473	0.810
Coriobacteriaceae	2.38	2.46	2.22	2.96	2.07	1.21	0.973	0.836
Staphylococcaceae	0.09	0.71	0.33	0.04	4.52	2.13	0.128	0.221
Leuconostocaceae	0.24	1.86	0.08	0.57	1.22	0.86	0.767	0.907
Burkholderiaceae	0.50	0.66	0.05	1.54	0.08	0.72	0.989	0.681
Peptostreptococcaceae	0.73	0.49	1.21	0.17	0.18	0.38	0.253	0.384
Others	6.22	13.15	6.40	7.84	5.04	4.26	0.578	0.491

^a^Experimental unit was a pig and level had 4 replicates (totaling 20 observations), representing the approximate median BW of each level

^b^*SEM* Standard error of the means

**Table 4:** Relative abundance of jejunal mucosa-associated microbiota at the genus level in nursery pigs fed diets with increasing levels of xylanase and β-glucanase combination (XG)^a^

Item	XG, (TXU/TGU)/kg					SEM^b^	*P* value	
	0	280/125	560/250	840/375	1,120/500		Linear	Quadratic
*Lactobacillus*	14.90	28.97	50.09	27.68	14.99	11.88	0.976	0.043
*Helicobacter*	36.09	4.49	6.40	17.01	43.67	12.54	0.497	0.018
*Bifidobacterium*	13.42	19.40	5.99	19.59	7.93	6.67	0.616	0.745
*Streptococcus*	2.87	9.03	6.78	3.70	3.29	2.86	0.537	0.123
*Olsenella*	1.89	1.69	1.47	2.63	1.71	1.05	0.866	0.989
*Megasphaera*	1.19	2.28	1.79	1.87	1.87	0.77	0.626	0.492
*Prevotella*	3.21	0.34	1.18	0.63	1.61	1.02	0.383	0.122
*Staphylococcus*	0.09	0.68	0.32	0.04	4.51	2.13	0.128	0.219
*Mitsuokella*	0.85	0.62	0.76	1.09	1.00	0.40	0.559	0.758
*Weissella*	0.24	1.86	0.08	0.57	1.21	0.86	0.766	0.907
*Blautia*	0.82	0.70	1.52	0.34	0.58	0.54	0.590	0.495
*Dialister*	0.97	0.48	0.75	0.73	0.86	0.35	0.978	0.489
*Faecalibacterium*	1.22	0.20	0.80	0.56	0.57	0.45	0.515	0.477
*Roseburia*	0.96	0.46	0.82	0.39	0.46	0.44	0.444	0.824
*Solobacterium*	0.79	0.56	0.52	0.81	0.34	0.27	0.457	0.892
*Ralstonia*	0.50	0.66	0.05	1.53	0.08	0.72	0.986	0.685
Others	19.97	26.16	20.68	19.40	16.74	6.49	0.530	0.587

^a^Experimental unit was a pig and each level had 4 replicates (totaling 20 observations), representing the approximate median BW of each level

^b^*SEM* Standard error of the means

**Table 5:** Relative abundance of jejunal mucosa-associated microbiota at the species level in nursery pigs fed diets with increasing levels of xylanase and β-glucanase combination (XG)^a^

Item	XG, (TXU/TGU)/kg					SEM^b^	*P* value	
	0	280/125	560/250	840/375	1,120/500		Linear	Quadratic
*Helicobacter rappini*	35.93	4.28	4.55	16.88	42.17	12.68	0.542	0.019
*Bifidobacterium dentium*	11.84	11.60	3.75	16.34	6.23	5.75	0.727	0.975
*Lactobacillus mucosae*	3.08	5.23	7.82	5.04	2.58	2.38	0.875	0.124
*Lactobacillus *sp.	3.86	4.56	5.34	4.65	2.16	2.37	0.664	0.391
*Lactobacillus delbrueckii*	3.41	3.84	5.00	4.66	1.87	2.16	0.747	0.342
*Lactobacillus delbrueckii*	3.41	3.84	5.00	4.66	1.87	2.16	0.747	0.342
*Lactobacillus salivarius*	0.44	2.99	11.60	0.32	0.30	5.07	0.857	0.208
*Lactobacillus johnsonii*	0.11	2.83	4.95	1.23	1.99	1.64	0.624	0.080
*Bifidobacterium boum*	0.38	6.97	1.52	1.16	0.76	2.47	0.528	0.352
*Megasphaera *sp.	1.14	2.26	1.75	1.84	1.81	0.75	0.622	0.453
*Olsenella profusa*	1.15	1.08	0.86	2.18	0.99	0.77	0.754	0.811
*Prevotella copri*	2.77	0.27	0.96	0.62	1.36	0.89	0.396	0.125
*Bifidobacterium thermacidophilum*	1.21	0.81	0.66	2.09	0.94	0.86	0.788	0.985
*Streptococcus hyointestinalis*	0.02	1.86	0.50	0.05	0.47	0.72	0.700	0.486
*Ralstonia pickettii*	0.50	0.66	0.05	1.53	0.08	0.72	0.986	0.685
*Streptococcus parasuis*	0.03	2.31	0.06	0.27	0.02	0.77	0.419	0.383
*Mitsuokella multacida*	0.67	0.46	0.45	0.45	0.66	0.31	0.959	0.467
*Helicobacter equorum*	0.16	0.03	0.83	0.13	1.50	0.56	0.137	0.486
*Faecalibacterium prausnitzii*	0.92	0.19	0.66	0.33	0.53	0.37	0.592	0.453
*Staphylococcus xylosus*	0.04	0.41	0.09	0.02	2.04	0.96	0.137	0.218
*Weissella thailandensis*	0.12	0.86	0.07	0.22	1.16	0.65	0.375	0.485
Others	30.61	38.54	44.32	29.78	29.06	8.70	0.664	0.256

^a^Experimental unit was a pig and each level had 4 replicates (totaling 20 observations), representing the approximate median BW of each level

^b^*SEM* Standard error of the means

### Immune responses and oxidative damage products in the jejunum

Increasing levels of XG tended to linearly decrease (*P* = 0.073) IgG contents in jejunal mucosa of pigs (Table 6). However, there was no difference with increasing levels of XG or XG intake in IgA, IL-8, TNF-α, MDA, and protein carbonyl.

**Table 6:** Immune responses and oxidative damage products from mid-jejunal mucosa of nursery pigs fed with increasing levels of xylanase and β-glucanase combination (XG)

Item, /mg of protein	XG, (TXU/TGU)/kg					SEM^b^	*P* value	
	0	280/125	560/250^a^	840/375	1,120/500		Linear	Quadratic
IgA^c^, µg	3.12	3.06	3.58	4.12	3.25	0.67	0.566	0.536
IgG^d^, µg	2.83	1.41	2.37	1.66	1.42	0.43	0.073	0.669
IL-8^e^, ng	0.51	0.34	0.49	0.46	0.45	0.08	0.981	0.668
TNF-α^f^, pg	6.11	5.15	5.15	7.24	5.48	1.05	0.804	0.900
MDA^g^, nmol	0.38	0.29	0.28	0.30	0.31	0.06	0.522	0.286
PC^h^, nmol	2.68	2.79	2.18	1.83	3.25	0.66	0.938	0.254

^a^An observation of pigs fed a diet with 560/250 (TXU/TGU)/kg was removed from the data set for the final analysis as the observation confirmed as an outlier; experimental unit was a pig and each level had 8 replicates (totaling 40 observations)

^b^*SEM* Standard error of the means

^c^*IgA* Immunoglobulin A

^d^*IgG* Immunoglobulin G

^e^*IL-8* Interleukin 8

^f^*TNF-α* Tumor necrosis factor alpha

^g^*MDA* Malondialdehyde

^h^*PC* Protein carbonyl

### Intestinal morphology and crypt cell proliferation in the jejunum

Increasing levels of XG tended to quadratically increase (*P* = 0.085) jejunal VH:CD reaching the maximum (2.62) at 560 TXU/kg and 250 TGU/kg (Table 7). However, there was no difference with increasing levels of XG or XG intake in VH, CD, and Ki-67^+^.

**Table 7:** Intestinal morphology and crypt cell proliferation of nursery pigs fed diets with increasing levels of xylanase and β-glucanase combination (XG)

Item	XG, (TXU/TGU)/kg					SEM^b^	*P* value	
	0	280/125	560/250^a^	840/375	1,120/500		Linear	Quadratic
Mid-jejunum								
Villus height, µm	492	553	525	515	535	26	0.572	0.530
Crypt depth, µm	214	220	208	198	232	11	0.692	0.166
VH:CD^c^	2.33	2.53	2.59	2.65	2.32	0.15	0.827	0.085
Ki-67^+d^, %	32.2	31.9	34.5	30.9	30.1	1.21	0.195	0.138

^a^An observation of pigs fed a diet with 560/250 (TXU/TGU)/kg was removed from the data set for the final analysis as the observation confirmed as an outlier; experimental unit was a pig and each level had 8 replicates (totaling 40 observations)

^b^*SEM* Standard error of the means

^c^*VH:CD* Villus height to crypt depth ratio

^d^Ratio of Ki-67 positive cell to total cell in the crypt, which represents crypt cell proliferation

### Apparent ileal digestibility of nutrients and energy

**Fig. 4:**
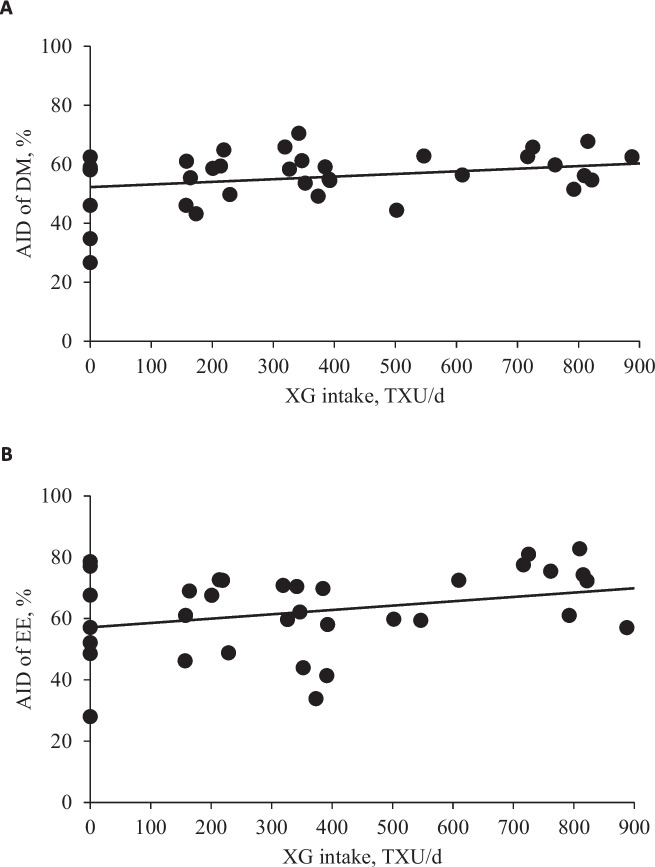
**A** Apparent ileal digestibility (AID) of dry matter (DM) in nursery pigs fed diets with increasing levels of xylanase and β-glucanase combination (XG) intake (TXU/d). AID of DM, % = 0.009 × XG intake + 52.25; *P* value of overall model: 0.087, *P* value of intercept: < 0.001, and *P* value of slope: 0.087. **B** Apparent ileal digestibility (AID) of ether extract (EE) in nursery pigs fed diets with increasing levels of xylanase and β-glucanase combination (XG) intake (TXU/d). AID of EE, % = 0.014 × XG intake + 57.13; *P* value of overall model: 0.065, *P* value of intercept: < 0.001, and *P* value of slope: 0.065. The ratio of TXU to TGU in XG was 2.24. The number of observation was 40

Increasing levels of XG tended to linearly increase (*P* = 0.072) the AID of DM (Table 8). Increasing XG intake tended to linearly increase the AID of DM (*P* = 0.087) and EE (*P* = 0.065, Fig. 4). However, there were no differences with increasing levels of XG or XG intake in AID of GE, CP, and EE.

**Table 8:** Apparent ileal digestibility of nutrients (DM basis) in nursery pigs fed diets with increasing levels of xylanase and β-glucanase combination (XG)

Item	XG, (TXU/TGU)/kg					SEM^b^	*P* value	
	0	280/125	560/250^a^	840/375	1,120/500		Linear	Quadratic
Dry matter, %	49.3	54.8	59.3	56.3	58.2	3.2	0.072	0.246
Gross energy, %	59.0	63.5	66.0	63.5	65.1	2.7	0.187	0.315
Crude protein, %	60.5	69.2	69.0	62.0	67.4	2.6	0.444	0.208
Ether extract, %	58.4	62.5	58.1	62.1	70.6	5.0	0.137	0.361

^a^An observation of pigs fed a diet with 560/250 (TXU/TGU)/kg was removed from the data set for the final analysis as the observation confirmed as an outlier; experimental unit was a pig and each level had 8 replicates (totaling 40 observations)

^b^*SEM* Standard error of the means

### Growth performance and fecal score

**Fig. 5:**
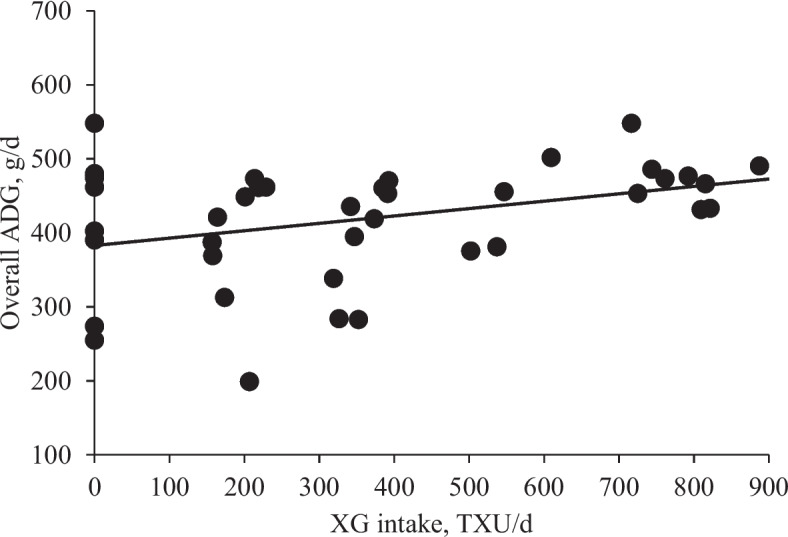
Average daily gain (ADG) of nursery pigs fed diets with increasing levels of xylanase and β-glucanase combination (XG) intake (TXU/d). Overall ADG, g/d = 0.09 × XG intake + 392; *P* value of overall model: 0.021, *P* value of intercept: < 0.001, and *P* value of slope: 0.021. The ratio of TXU to TGU in XG was 2.24. The number of observation was 39 excluding one outlier fed a diet with 560/250 (TXU/TGU)/kg following the suggestion from the IACUC

Increasing levels of XG did not affect the fecal score and growth performance including ADG, ADFI, and G:F of nursery pigs (Tables 9 and 10). Increasing XG intake linearly increased (*P* < 0.05) ADG (Fig. 5).

**Table 9:** Fecal score of nursery pigs fed diets with increasing levels of xylanase and β-glucanase combination (XG)

Item	XG, (TXU/TGU)/kg					SEM^b^	*P* value	
	0	280/125	560/250^a^	840/375	1,120/500		Linear	Quadratic
Phase 1 (d 0 to 11)	3.95	4.13	3.99	3.99	4.01	0.12	0.970	0.705
Phase 2 (d 11 to 20)	3.24	3.12	3.17	3.18	3.12	0.07	0.398	0.761
Phase 3 (d 20 to 35)	3.15	3.13	3.10	3.14	3.11	0.03	0.721	0.762

^a^An observation of pigs fed a diet with 560/250 (TXU/TGU)/kg was removed from the data set for the final analysis as the observation confirmed as an outlier; experimental unit was a pig and each level had 8 replicates (totaling 40 observations)

^b^*SEM* Standard error of the means

**Table 10:** Growth performance of nursery pigs fed diets with increasing levels of xylanase and β-glucanase combination (XG)

Item	XG, (TXU/TGU)/kg					SEM^b^	*P* value	
	0	280/125	560/250^a^	840/375	1,120/500		Linear	Quadratic
Body weight, kg								
Day 0	6.5	6.5	6.5	6.5	6.5	0.4	1.000	0.807
Day 11	7.2	7.4	7.2	7.4	7.7	0.6	0.269	0.542
Day 20	11.0	10.9	11.4	11.4	11.6	0.9	0.257	0.868
Day 35	20.8	21.1	21.3	21.0	22.7	1.4	0.204	0.456
Average daily gain, g/d								
Phase 1 (d 0 to 11)	69	84	67	82	112	24	0.215	0.449
Phase 2 (d 11 to 20)	423	385	465	443	439	38	0.396	0.783
Phase 3 (d 20 to 35)	654	680	657	641	740	43	0.246	0.268
Overall (d 0 to 35)	411	417	422	414	465	32	0.183	0.424
Average daily feed intake, g/d								
Phase 1 (d 0 to 11)	162	173	152	155	202	24	0.301	0.191
Phase 2 (d 11 to 20)	598	561	643	638	643	72	0.388	0.987
Phase 3 (d 20 to 35)	985	1,114	1,010	1,009	1,164	78	0.165	0.475
Overall (d 0 to 35)	627	676	646	645	727	57	0.200	0.546
Gain to feed ratio								
Phase 1 (d 0 to 11)	0.06	0.46	0.41	0.39	0.54	0.24	0.181	0.564
Phase 2 (d 11 to 20)	0.74	0.70	0.73	0.73	0.69	0.04	0.469	0.847
Phase 3 (d 20 to 35)	0.67	0.62	0.65	0.65	0.64	0.02	0.695	0.585
Overall (d 0 to 35)	0.66	0.62	0.65	0.65	0.64	0.02	0.918	0.724

^a^An observation of pigs fed a diet with 560/250 (TXU/TGU)/kg was removed from the data set for the final analysis as the observation confirmed as an outlier; experimental unit was a pig and each level had 8 replicates (totaling 40 observations)

^b^*SEM* Standard error of the means

## Discussion

Corn DDGS became a commodity feedstuff because of the availability and economic advantages [1]. However, high NSP contents in corn DDGS negatively affect the intestinal health and growth of nursery pigs [3, 4]. High NSP in feeds leads to increased digesta viscosity in the small intestine [2], and thus, reduced nutrient utilization by blocking the access of endogenous enzymes to ingested feeds in the small intestine of pigs [49]. In addition, increased digesta viscosity in the small intestine reduces passage rate of digesta and accelerates bacterial fermentation resulting in the overgrowth of potentially pathogenic bacteria, and thereby increasing intestinal inflammation and impairing growth of nursery pigs [8, 9]. The process of ethanol production from corn includes soaking, heating, enzymatic digestion, and yeast fermentation making NSP in corn soluble, and thus corn DDGS consists of higher soluble NSP fractions than corn which include arabinoxylans and β-glucans [50]. Feeds with high soluble NSP cause an increase in the viscosity of digesta due to their high water-holding capacity [2, 51]. However, arabinoxylans and β-glucans in feeds contain β-glycosidic linkages, requiring exogenous NSP-degrading enzymes for the degradation of these NSP fractions [52, 53]. Recently, NSP-degrading enzymes have shown synergistic effects on the hydrolysis of NSP, effectively decreasing the digesta viscosity in the small intestine in relation to enhanced intestinal health and growth of nursery pigs [3, 30, 54].

Xylanase supplementation has been shown to decrease the viscosity of jejunal digesta in nursery pigs [3, 13]. This reduction in viscosity is likely attributed to the breakdown of soluble NSP fractions. The NSP-degrading enzymes effectively degraded soluble NSP in the small intestine of pigs, but little degradation was observed in insoluble NSP [55]. The degree of improvement in AID of soluble NSP by xylanase was greater than that of insoluble NSP in pigs [7]. β-glucanase also decreased digesta viscosity more effectively with soluble β-glucans than insoluble β-glucans in the feeds [24]. According to Li et al. [18], the β-glucanase improved the β-glucan utilization in the small intestine, but no difference was observed in the large intestine of nursery pigs.

The results of this study indicate that XG has a relatively higher influence on the degradation of soluble NSP fractions than insoluble NSP in the small intestine of pigs, reducing digesta viscosity. However, this study showed a quadratic change of digesta viscosity as XG supplementation increased, indicating that high level XG supplementation could increase the digesta viscosity of pigs. There is a possibility that the structure of insoluble NSP is loosened by these enzymes making NSP relatively soluble. Soluble NSP fractions have open molecular structures [14], which can be more susceptible to NSP-degrading enzymes [7, 56]. Previous studies have reported that high levels of xylanase supplementation increased soluble NSP contents [23, 30, 54], leading to an increase in the viscosity of digesta in the small intestine in pigs [21, 52] and chickens [57, 58].

Linear increases in the AID of DM and EE were observed with increasing XG intake. The reason for a linear improvement in AID of EE with increasing XG intake could be partly due to the release of entrapped fats by viscose soluble NSP, as well as the release of bile salts bound to soluble NSP. With the supplementation of xylanase, bile salts bound to the soluble NSP fraction were released, improving lipid digestion and absorption in the small intestine of pigs [13, 59]. Additionally, xylan and fat contents are greater in the pericarp compared with the endosperm in cell wall of corn and corn byproducts [60, 61], potentially improving AID of EE by XG in this study.

Investigation of NSP-degrading enzymes has gained attention for their functional roles in benefitting intestinal health and intestinal microbiota of pigs [13, 20, 62, 63]. In this study, an increase in beneficial intestinal bacteria, such as *Lactobacillus*, and a decrease in opportunistic pathogenic bacteria such as *Helicobacter rappini* in the jejunal mucosa were observed with XG. This may be due to the reduction of digesta viscosity. Previously, it was shown that pigs with increased digesta viscosity in the small intestine had increased population of *Escherichia coli* [9, 64]. Increased relative abundance of *Helicobacter rappini* in the jejunal mucosa had negative effects on the intestinal health and growth of nursery pigs [10, 65]. It was shown that a combinational use of xylanase and β-glucanase reduced the relative abundance of *Helicobacter rappini* in the jejunal mucosa of nursery pigs [20]. Xylanase supplementation has been shown to positively modulate the mucosa-associated microbiota, including an increase in *Succinivibrio*, as it reduced digesta viscosity in the jejunum of nursery pigs [13].

It has been shown that xylanase and β-glucanase effectively hydrolyze arabinoxylans and β-glucans in various feedstuffs releasing xylooligosaccharides [14, 15] and glucooligosaccharides [19] in vitro. There are several in vivo studies demonstrating increased release of oligosaccharides by the supplementation of xylanase [13, 52, 62] and β-glucanase [24, 66] through the hydrolysis of NSP fractions providing prebiotic substrates for beneficial bacteria in the small intestine of pigs. Prebiotic effects of these oligosaccharides could be another factor for the positive modulation of mucosa-associated microbiota and improvement of the intestinal health and growth of nursery pigs as observed in this study.

*Lactobacillus*, considered a beneficial bacteria, is known to produce lactic acid from fermentable NSP in the gastrointestinal tract of pigs [67]. Xylooligosaccharides in nursery feeds have been shown to increase the abundance of *Lactobacillus* and reduce the abundance of *E. coli* in feces in relation to improved growth of nursery pigs [68]. The relative abundance of Lactobacillaceae was increased at the XG supplementation was increased until 608 TXU/kg with 271 TGU/kg and then started to decrease when the supplementation was further increased in this study. Interestingly, with the supplementation of XG, a quadratic change was observed in the relative abundance of Prevotellaceae, a fibrolytic bacteria in the jejunal mucosa. An increase of the abundance of fibrolytic bacteria is related to the increased production of short-chain fatty acids [69], showing beneficial effects on intestinal health of pigs [46].

The possible reason for this may be partly due to the decrease in substrates for Prevotellaceae, such as cellulose and xylans, caused by NSP-degrading enzymes [70]. However, in a previous study, xylanase supplementation increased *Prevotella*, which belongs to Prevotellaceae, in the feces [71]. The deviations between the previous study and this study may be due to the difference in the location of microbiota within the intestine (small intestine vs. large intestine) [72], the types of microbiota (mucosa-associated vs. luminal) [10, 11], and the types of feedstuffs (corn DDGS vs. wheat DDGS) [30]. This result suggests that there can be an optimal supplementation level of NSP-degrading enzymes for the microbiota in the jejunal mucosa of pigs whereas nutrient digestibility and intestinal health were continuously improved. Similarly, the relative abundance of Lactobacillaceae and Helicobacteraceae showed quadratic changes indicating similar optimal levels of XG. The pattern of microbial changes in the jejunal mucosa was closely related to the changes in digesta viscosity. Increase in digesta viscosity reduces the nutrient availability for intestinal microbiota and inhibits the attachment and proliferation of intestinal microbiota using available nutrients. Tamargo et al. [73] showed that the intestinal microbiota is influenced not only by NSP contents but also by digesta viscosity.

The supplementation of XG had a positive impact on intestinal immune response, intestinal morphology parameters, and growth of nursery pigs in this study. The reason for this may result from the positive modulation of microbiota in the jejunal mucosa by the release of oligosaccharides [68, 74, 75], as well as an increase in nutrient availability with a reduction of digesta viscosity [10, 13, 53].

Mucosa-associated microbiota plays an important role in maintaining and improving intestinal health in pigs as mucosa-associated microbiota have close physical contact and biological communication with the host intestinal cells [10, 11, 76] affecting intestinal immune system and the ability to resist to pathogenic colonization [77].

Some previous studies reported that xylanase supplementation can attenuate oxidative stress reducing the oxidative damage product including MDA in the jejunum of nursery pigs [3, 13]. However, another previous study reported no effects of xylanase on oxidative stress in the jejunum of nursery pigs [54]. The deviation between the previous studies [3, 13] and this study may be due to the low MDA in the jejunal mucosa of pigs fed control diets without XG, leaving little room for the reduction of MDA content in the jejunum with XG supplementation. However, the MDA content obtained in this study was within a typical value compared to other recent measurements from pigs with similar genetic background and feeding environment [29, 78–80].

## Conclusion

A combinational use of xylanase and β-glucanase would hydrolyze the non-starch polysaccharides fractions, positively modulating the jejunal mucosa-associated microbiota. Increased intake of these enzyme combination possibly reduced digesta viscosity and humoral immune response in the jejunum resulting in improved intestinal structure, and ileal digestibility of nutrients, and finally improving growth of nursery pigs. The beneficial effects were maximized at a combination of 550 to 800 TXU/kg xylanase and 250 to 360 TGU/kg β-glucanase.

## Data Availability

All data generated or analyzed during this study are available from the corresponding author upon reasonable request.

## Abbreviations

ADFI: Average daily feed intake
ADG: Average daily gain
AID: Apparent ileal digestibility
ASV: Amplicon sequence variant
BW: Body weight
CD: Crypt depth
CP: Crude protein
DDGS: Distiller’s dried grains with solubles
DM: Dry matter
G:F: Gain to feed ratio
EE: Ether extract
GE: Gross energy
IgA: Immunoglobulin A
IgG: Immunoglobulin G
IL-8: Interleukin-8
MDA: Malondialdehyde
NSP: Non-starch polysaccharides
SID: Standardized ileal digestible
STTD: Standardized total tract digestible
TGU: Endo-1,4-β-glucanase activity
TiO_2_: Titanium dioxide
TNF-α: Tumor necrosis factor-alpha
TXU: Endo-1,4-β-xylanase activity
VH: Villus height
VH:CD: Villus height to crypt depth ratio
XG: Xylanase and β-glucanase combinations
